# Survival, growth and sexual maturation in Atlantic salmon exposed to infectious pancreatic necrosis: a multi-variate mixture model approach

**DOI:** 10.1186/1297-9686-45-8

**Published:** 2013-03-25

**Authors:** Marie Lillehammer, Jørgen Ødegård, Per Madsen, Bjarne Gjerde, Terje Refstie, Morten Rye

**Affiliations:** 1 Nofima, P.O. Box 210, Ås, NO-1431, Norway; 2 Department of Molecular Biology and Genetics, Center for Quantitative Genetics and Genomics, Research Centre Foulum, University of Aarhus, Blichers Allé, P.O. Box 50, Tjele, DK-8830, Denmark; 3 Akvaforsk Genetics Center, Sjølseng, Sunndalsøra, N-6600, Norway

## Abstract

**Background:**

Outbreaks of infectious pancreatic necrosis (IPN) in Atlantic salmon can result in reduced growth rates in a fraction of the surviving fish (runts). Genetic and environmental variation also affects growth rates within different categories of healthy animals and runts, which complicates identification of runts. Mixture models are commonly used to identify the underlying structures in such data, and the aim of this study was to develop Bayesian mixture models for the genetic analysis of health status (runt/healthy) of surviving fish from an IPN outbreak.

**Methods:**

Five statistical models were tested on data consisting of 10 972 fish that died and 3959 survivors with recorded growth data. The most complex models (4 and 5) were multivariate normal-binary mixture models including growth, sexual maturity and field survival traits. Growth rate and liability of sexual maturation were treated as two-component normal mixtures, assuming phenotypes originated from two potentially overlapping distributions, (runt/normal). Runt status was an unobserved binary trait. These models were compared to mixture models with fewer traits (Models 2 and 3) and a classical linear animal model for growth (Model 1).

**Results:**

Assuming growth as a mixture trait improved the predictive ability of the statistical model considerably (Model 2 vs. 1). The final models (4 and 5) yielded the following results: estimated (underlying) heritabilities were moderate for growth in healthy fish (0.32 ± 0.04 and 0.35 ± 0.05), runt status (0.39 ± 0.07 and 0.36 ± 0.08) and sexual maturation (0.33 ± 0.05), and high for field survival (0.47 ± 0.03 and 0.48 ± 0.03). Growth in healthy animals, runt status and survival showed consistent favourable genetic associations. Sexual maturation showed an unfavourable non-significant genetic correlation with runt status, but favourable genetic correlations with other traits. The estimated fraction of healthy fish was 81-85%. The estimated breeding values for runt status and (normal) growth were consistent for the most complex models (4 and 5), but showed imperfect correlations with estimated breeding values from the simpler models.

**Conclusions:**

Modelling growth in IPN survivors as a mixture trait improved the predictive ability of the model compared with a classical linear model. The results indicated considerable genetic variation in health status among survivors. Mixture modelling may be useful for the genetic analysis of diseases detected mainly through indicator traits.

## Background

Infectious pancreatic necrosis (IPN) is a viral disease affecting salmonid fishes. Survival after exposure to IPN virus is highly heritable in Atlantic salmon [1-3], where the majority of genetic variation is controlled by a single major QTL [4,5]. Under field conditions, infection with IPN virus not only causes mortality, but also poor growth of some of the affected survivors, so-called “IPN runts” [6,7]. In field data, the true health status of surviving fish is usually unknown, thus the growth of fish in a population containing IPN runts can be considered as a variable drawn from a mixture distribution with (at least) two sub-distributions. Each sub-distribution represents different health classes (e.g., healthy fish and IPN runts). Finite mixture models are useful in situations where an underlying group structure exists (e.g., unknown health status of fish) in the data [8], and can be used to obtain information on individual health status based only on observed growth (and potentially other indicator traits). Previously, mixture models have been used to analyse milk somatic cell count data [9-12] and better understand subclinical mastitis in dairy cows. Using the model described by Ødegård et al. [10], the health status of fish may be considered as an unobserved binary variable affecting the distribution of growth data in Atlantic salmon exposed to IPN. Probability of these hidden binary classes can be modeled through a liability-normal mixture model. Thus, data on growth can contribute to breeding values for health, even in the absence of direct recording of the disease (given that the fish have been exposed to infection). Furthermore, since the growth of IPN runts does not reflect their true growth potential in a disease-free environment, the mixture model can be used to predict more reliable breeding values for growth potential, even based on data from disease-exposed environments.

The main objective of this study was to apply a mixture model to two traits, i.e. growth and sexual maturation, the latter being a binary trait (sexual maturation). A secondary objective was to estimate genetic parameters for health and correlated traits from surviving fish after a natural outbreak of IPN in a commercial production environment of Atlantic salmon.

## Methods

### Data

Phenotypic data on survival, growth, sex and sexual maturation of Atlantic salmon were obtained from the 2002 year-class of the Salmobreed population. Fish were the offspring from a hierarchical mating design in which one sire was mated with two dams and each dam to one sire only. The pedigree used for the genetic analyses consisted of the recorded fish and their parents (152 sires and 294 dams), the latter coming from a non-pedigreed farmed salmon population. The tagged individuals were random samples from each family among the individuals that had reached tagging size. Families were kept separate until tagging before all tagged individuals were transferred to a single sea cage. The fish (N = 14 931 with average tagging weight of 31.8 g (SD = 8.1 g), tagged in October-November 2002) in this year-class suffered very high mortalities (72%) due to a natural outbreak of IPN during the first summer at sea. Since dead fish were not individually tested for IPN, a fraction (probably quite small) of the mortality may have had other causes. The surviving fish were slaughtered or selected as breeders during three different periods between March 21 and August 24, 2005. The number of fish from each sex-maturation class and their average weights at the different harvesting times are summarized in Table 1, which shows that harvesting time was not random (a result of practical considerations within the breeding nucleus). Growth potential was assessed by growth rate (GR), calculated as: GR = (harvest weight (g) – tagging weight (g))/days between tagging and harvest, probably a more robust measure than the crude weight of the fish.

**Table 1:** Number of fish from each sex-maturation class harvested at each harvest time

	**Males**		**Females**	
**Harvesting time**	**Non-maturing**	**Maturing**	**Non-maturing**	**Maturing**
March 21-22	-	240 (10.7)	-	-
June 29	1289 (5.1)	54 (5.7)	925 (3.6)	82 (5.5)
August 23-24	465 (8.8)	138 (9.6)	128 (7.9)	639 (8.1)

Average harvest weight (kg) is given in brackets.

Furthermore, the sex (male/female) and sexual maturation status (maturing = 1/not maturing = 0) of each fish were recorded, based on external characteristics. Growth rates of fish of unknown sex (N = 5) and fish with external characteristics indicating that they had reached sexual maturity the year before, defined as early sexual mature (N = 232), were not included in the statistical analysis of growth, but these fish were included as survivors (for the analyses including survival). This resulted in 3959 growth and 14 931 survival (dead = 0/alive = 1) records. This data has not been used in previous publications.

### Statistical modeling

A preliminary analysis of growth (results not shown) using standard linear models indicated a non-significant effect common to full-sibs (e.g., due to separate rearing of each full-sib family until tagging), in addition to additive genetics, and this effect was thus not included in the final statistical models.

In the mixture models described here, observations in the data vector **y**_**1**_ (growth data) are assumed to come from two overlapping distributions (mixture components); one distribution for growth of healthy animals, and one distribution for growth of infected animals (runts). The two mixture components are assumed to have different means (runts are assumed to have a lower mean), and potentially different variances. The sub-distribution to which each observation belongs and parameters of the two sub-distributions are assumed unknown, i.e., health status of animals is assumed unknown and must be inferred from the observed growth data. Following the notation in Ødegård et al. [9], putative health statuses of all fish are included in a vector **z**, which has one element for each growth observation in the dataset. If observation *i* is assumed to come from a healthy animal, the corresponding element in **z**, *z*_*i*_ = 1, otherwise *z*_*i*_ = 0. Furthermore, we define a diagonal matrix **M**_**z**_ = diag(**z**). The fully conditional densities of the individual mixture variables are proportional to independent binary distributions with a success (healthy) probability:

$$\tau_{i}=\frac{p\left(y_{1i}\left|\mathrm{model},z_{i}=1\right)\right)\cdot \mathrm{Pr}\left(z_{i}=1\left|\mathrm{model}\right)\right)}{p\left(y_{1i}\left|\mathrm{model},z_{i}=0\right)\right)\cdot \mathrm{Pr}\left(z_{i}=0\left|\mathrm{model}\right)\right)+p\left(y_{1i}\left|\mathrm{model},z_{i}=1\right)\right)\cdot \mathrm{Pr}\left(z_{i}=1\left|\mathrm{model}\right)\right)},$$

where p(*y*_1*i*_|model, *z*_*i*_ = 1) is the likelihood of the observation (under the current model parameters), given that the animal is healthy, while p(*y*_1*i*_|model, *z*_*i*_ = 0) is the corresponding likelihood, given that the animal is diseased. Finally, Pr(*z*_*i*_ = 1|model) is the prior probability of being healthy, given the model, and Pr(*z*_*i*_ = 0|model) = 1 - Pr(*z*_*i*_ = 1|model).

Five statistical models were used to analyze the data. Model 1 was a classical linear (non-mixture) animal model for genetic analysis of growth in salmon, while Models 2–5 were mixture animal models of varying complexity and considering different traits.

#### Model 1: Classical animal model for growth

$$y_{1}=X_{10}\beta_{10}+Z_{a}a_{1}+e_{1},$$

where **y**_**1**_ is a vector of observed growth rates, **β**_**10**_ is a vector including fixed effects of sex by sexual maturation; $a_{1}\sim N\left(0,A\sigma_{a}^{2}\right)$, with **a**_**1**_ being a vector of additive genetic effects of growth for all animals and **A** is the numerator relationship matrix, $e_{1}\sim N\left(0,I_{n}\sigma_{e}^{2}\right)$, with **e**_**1**_ being a vector of random residuals; where **I**_***n***_ is an identity matrix of dimension *n* (number of animals with growth-data); and the **X** and **Z** matrices are appropriate incidence matrices.

#### *Model 2: Bivariate animal mixture model for growth and health status*

$$y=\left[\begin{array}{c} y_{1}\\ \lambda_{2} \end{array}\right]=[\begin{array}{c} X_{10}\beta_{10}+M_{z}X_{11}\beta_{11}+M_{z}Z_{a}a_{1}+e_{1}\\ X_{20}\beta_{20}+Z_{a}a_{2}+e_{2} \end{array}\;],$$

where **λ**_**2**_ is a vector of unobserved liabilities associated with the mixture variable vector **z,** i.e. the diagonal elements of **M**_**z**_, also unobserved; if an element of this vector takes the value *z*_*i*_ = 1, this implies that the associated liability *λ*_*2i*_ ≥ 0, while *z*_*i*_ = 0 implies that *λ*_*2i*_ < 0 (as in a standard probit threshold model). The factor **β**_**10**_ is now a vector including the fixed sex- and maturation-specific means for growth rate of runts; **β**_**11**_ is a vector of fixed effects means for growth rates of healthy animals, as deviations from the mean growth rate of runts, nested within three categories: maturing fish, non-maturing males and non-maturing females (distinguishing health effects of males and females within maturing fish caused estimation problems, since practically no maturing females were classified as runts); **X**_**11**_ is a design matrix that assigns records to these three sex-maturation class categories; **β**_**20**_ is the overall mean of liabilities associated with health status; **X**_**20**_ is a vector of ones;

$$\begin{array}{ll} \;e & =\left[\begin{array}{c} e_{1}\\ e_{2} \end{array}\right]\sim \text{N}\left(0,R\right),R=\left[\begin{array}{cc} R_{1} & 0\\ 0 & I_{n} \end{array}\right],\\ \;R_{1} & =M_{z}\sigma_{\mathrm{eH}}^{2}+\left(I_{n}-M_{z}\right)\sigma_{\mathrm{eD}}^{2}, \end{array}$$

where $\sigma_{\mathrm{eH}}^{2}$ is the residual variance of growth for healthy animals, while $\sigma_{\mathrm{eD}}^{2}$ is the residual variance of growth in runts.

Other factors are as in model 1. In model 2, growth potential was assumed to be expressed in healthy animals only.

$$a=\left[\begin{array}{c} a_{1}\\ a_{2} \end{array}\right]\sim \text{N}\left(0,G⊗A\right),$$

where **G** is the additive genetic (co)variance matrix of growth and health status liability and **G⊗A** is the Kronecker product of **G** and the additive relationship matrix **A**.

#### Model 3: Multivariate animal mixture model for growth, health status and sexual maturation

Analysis of the results of the models described above, and of the plots of the observed growth phenotypes for the different sex by maturation classes, showed that the frequency of runts differed among classes. Based on visual plots and outputs from the models, it was clear that the number of runts was lower among sexually maturing fish. This may be due to delayed sexual maturation in fish that had undergone infection (potentially as a result of retarded growth and reduced body reserves). Furthermore, sexual maturation itself also affects growth (fish that begin to mature are probably under hormonal stimulation of growth). Thus, in the models described above, there may be a confounding effect between health status and sexual maturation on growth. For this reason, a third model was developed, in which health status was also assumed to affect the liability for sexual maturation. Thus, a healthy fish was assumed to have both increased liability for sexual maturation and increased growth, compared with a runt. This was modeled by including a third model with sexual maturation as the dependent variable. The sub-model for sexual maturation may be considered as a probit of mixtures [13], and the full model may be written as:

$$y=\left[\begin{array}{c} y_{1}\\ \lambda_{2}\\ \lambda_{3} \end{array}\right]=[\begin{array}{c} X_{10}\beta_{10}+M_{z}X_{11}\beta_{11}+M_{z}Z_{a}a_{1}+e_{1}\\ X_{20}\beta_{20}+Z_{a}a_{2}+e_{2}\\ X_{30}\beta_{30}+M_{z}X_{31}\beta_{31}+Z_{a}a_{3}+e_{3} \end{array}\;],$$

where **λ**_**3**_ is a vector of unobserved liabilities associated with the observed sexual maturation status (non-maturing = 0, maturing =1) vector **m**; *m*_*i*_ = 1 implies that *λ*_*3i*_ ≥ 0, and *m*_*i*_ = 0 implies that *λ*_*3i*_ < 0, as in a standard probit threshold model; **β**_**30**_ includes the fixed effects of sex on the liability for sexual maturation, while **β**_**31**_ includes fixed sex-specific effects of health status on liability for sexual maturation, $a=\left[\begin{array}{c} a_{1}\\ a_{2}\\ a_{3} \end{array}\right]\sim N\left(0,G⊗A\right)$, where **G** is the additive genetic (co)variance matrix of growth, liability to be healthy and liability to sexual maturation. Furthermore, the residuals were assumed distributed as:

$$e=\left[\begin{array}{c} e_{1}\\ e_{2}\\ e_{3} \end{array}\right]\sim N\left(0,R\right),\mathrm{where}\;R=\left[\begin{array}{ccc} R_{1} & & 0\\ & I_{n} & \\ 0 & & I_{n} \end{array}\right].$$

The fully conditional probability of the individual mixture variable *z*_*i*_ = 1 is now:

$$\tau_{i}=\frac{p\left(y_{1i},\lambda_{3i}\left|\mathrm{model},z_{i}=1\right)\right)\cdot \mathrm{Pr}\left(z_{i}=1\left|\mathrm{model}\right)\right)}{p\left(y_{1i},\lambda_{3i}\left|\mathrm{model},z_{i}=0\right)\right)\cdot \mathrm{Pr}\left(z_{i}=0\left|\mathrm{model}\right)\right)+p\left(y_{1i},\lambda_{3i}\left|\mathrm{model},z_{i}=1\right)\right)\cdot \mathrm{Pr}\left(z_{i}=1\left|\mathrm{model}\right)\right).}$$

Here, p(*y*_1*i*_, *λ*_3*i*_|model, *z*_*i*_ = 1) is the joint likelihood of growth and the current sample of the liability for sexual maturation, given that the animal is healthy, while p(*y*_1*i*_, *λ*_3*i*_|model, *z*_*i*_ = 0) is the corresponding liability given that the animal is infected.

#### Model 4: Multivariate animal mixture model for growth, health status, sexual maturation and survival

Due to the fact that most fish died prior to weight recording, and mortality was mainly due to IPN, for which resistance is highly heritable, the survivors may be viewed as a selected sample, and hence a multivariate model including survival would be appropriate:

$$y=\left[\begin{array}{c} \begin{array}{c} y_{1}\\ \lambda_{2} \end{array}\\ \begin{array}{c} \lambda_{3}\\ \lambda_{4} \end{array} \end{array}\right]=[\begin{array}{c} \begin{array}{c} X_{10}\beta_{10}+M_{z}X_{11}\beta_{11}+M_{z}Z_{a}a_{1}+e_{1}\\ X_{20}\beta_{20}+Z_{a}a_{2}+e_{2} \end{array}\\ \begin{array}{c} X_{30}\beta_{30}+M_{z}X_{31}\beta_{31}+Z_{a}a_{3}+e_{3}\\ X_{40}\beta_{40}+Z_{a}a_{4}+e_{4} \end{array} \end{array}\;],$$

where **λ**_**4**_ is a vector of liabilities associated with observed survival (vector **s**); *s*_*i*_ = 1 implies that *λ*_*4i*_ ≥ 0, and s_*i*_ = 0 implies that *λ*_*4i*_ < 0, as in a standard probit threshold model; **β**_**40**_ includes the overall mean for the underlying liability to survival;

$$a=\left[\begin{array}{c} a_{1}\\ a_{2}\\ a_{3}\\ a_{4} \end{array}\right]\sim \text{N}\left(0,G⊗A\right),$$

where **G** is the additive genetic (co)variance matrix of growth, liability to be healthy, liability for sexual maturation, and liability for survival;

$$\begin{array}{l} e=\left[\begin{array}{c} \begin{array}{c} e_{1}\\ e_{2} \end{array}\\ \begin{array}{c} e_{3}\\ e_{4} \end{array} \end{array}\right]\sim N\left(0,R\right),\\ R=\left[\begin{array}{cc} \begin{array}{cc} \begin{array}{c} \begin{array}{c} R_{1} \end{array}\\ \begin{array}{c} 0 \end{array} \end{array} & \begin{array}{c} \begin{array}{c} I_{n} \end{array} \end{array} \end{array} & \begin{array}{cc} \begin{array}{c} \begin{array}{c} I_{n} \end{array} \end{array} & \begin{array}{c} \begin{array}{c} 0 \end{array}\\ \begin{array}{c} I_{N} \end{array} \end{array} \end{array} \end{array}\right], \end{array}$$

where **I**_***N***_ is an identity matrix of dimension *N* (number of animals with survival data), and the other parameters are as defined above. Although survival was recorded on all fish, the other traits (i.e., growth rate, sexual maturation and health status) could only be recorded (or inferred) on survivors. For this reason, residual covariances between survival and the other traits were not estimable and were thus restricted to be zero.

#### Model 5: Multivariate animal mixture model for growth, health status, sexual maturation and survival, including genetic effect on growth in runts

This model investigated the presence of genetic effects on growth in runts, assuming that growth in healthy fish and growth in runts are potentially distinct genetic traits by distinguishing between a common genetic effect for growth expressed in all fish and a growth effect only expressed in healthy animals. The model was:

$$\begin{array}{ll} \;y & =\left[\begin{array}{c} y_{1}\\ \lambda_{2}\\ \lambda_{3}\\ \lambda_{4} \end{array}\right]\\ & =\left[\begin{array}{c} X_{10}\beta_{10}+M_{z}X_{11}\beta_{11}+Z_{a}a_{0}+M_{z}Z_{a}a_{1}+e_{1}\\ X_{20}\beta_{20}+Z_{a}a_{2}+e_{2}\\ X_{30}\beta_{30}+M_{z}X_{31}\beta_{31}+Z_{a}a_{3}+e_{3}\\ X_{40}\beta_{40}+Z_{a}a_{4}+e_{4} \end{array}\right]. \end{array}$$

Now,

$$a=\left[\begin{array}{c} a_{0}\\ a_{1}\\ a_{2}\\ a_{3}\\ a_{4} \end{array}\right]\sim N\left(0,G⊗A\right),$$

where **a**_**0**_ is the basic additive genetic effects on growth for all fish (runts and healthy), **a**_**1**_ is now the additional additive genetic effect on growth for healthy fish, **G** is the additive genetic (co)variance matrix of basic growth, additional growth in healthy fish, liability to be healthy, liability for sexual maturation, and liability for survival. Other parameters were defined above. Thus, this model included a new genetic effect, later referred to as ‘runt growth’, with an additive genetic variance equal to the variance in **a**_**0**_, which represents the growth potential given that an animal is a runt and can be seen as a measurement of the severity of the symptoms of infection.

### Bayesian structure and Gibbs sampling

All models were analyzed using the Gibbs sampling module (RJMC) of the DMU software package [14]. Genetic (co)variance components were sampled using an algorithm allowing the proper estimation of additive genetic (co)variance components for cross-sectional animal threshold models [15], i.e., based only on the additive genetic effects sampled for the parents, rather than for all animals in the pedigree. In general, conditional on **M**_**z**_, all models are reduced to standard multivariate models for binary and normally distributed traits. We used the standard prior distributions for all traits (i.e., growth and the underlying liability traits were all assumed to be Gaussian), liability traits had a residual variance equal to 1. The threshold value was assumed to be zero, implying that binary categories of 0 and 1 corresponded to positive and negative liabilities, respectively.

In the following, **α** = [**β**,**a**,**G**,**R**], **y**_**obs**_ is a vector of all observations (Gaussian and binary), **bin** is a vector of the binary observations only, and **λ** is a vector of the liabilities associated with all binary traits. The increased joint posterior density of all unknowns is then:

$$\begin{array}{ll} \;\text{p} & \left(\lambda ,\alpha ,z\left|y_{\mathrm{obs}}\right)\right)\propto \text{p}\left(y_{\mathrm{obs}}\left|\lambda \right),z,\alpha \right)\text{p}\left(\lambda ,z,\alpha \right)\\ & =\text{p}\left(y_{1}\left|z,\alpha \right)\right)\mathrm{Pr}\left(\mathrm{bin}\left|\lambda \right)\right)\text{p}\left(\lambda ,z\left|\alpha \right)\right)\text{p}\left(\alpha \right). \end{array}$$

The probability Pr(**bin**|**λ**) ensures that the standard assumptions for threshold models are fulfilled, i.e., that 0/1 observations correspond to negative/positive liabilities, respectively. Furthermore, in the most complex models (Models 4 and 5):

$$\begin{array}{ll} \;\text{p}\left(\lambda ,z\left|\alpha \right)\right) & =\text{p}\left(\lambda_{2},\lambda_{3},\lambda_{4},z\left|\alpha \right)\right)=\text{p}\left(\lambda_{3},\lambda_{4}\left|\lambda_{2},z,\alpha \right)\right)\text{p}\left(\lambda_{2},z\left|\alpha \right)\right)\\ & =\text{p}\left(\lambda_{3}\left|z,\alpha \right)\right)\left(\lambda_{4}\left|\alpha \right)\right)\text{p}\left(\lambda_{2}\left|\alpha \right)\right)\text{Pr}\left(z\left|\lambda_{2}\right)\right). \end{array}$$

The latter is true due to the fact that all traits are assumed conditionally independent (due to the structure of **R)**. Furthermore, conditioning on **z** is not relevant for **λ**_4_, as the model for this trait does not include **z**. The probability Pr(**z**|**λ**_**2**_) is equivalent to the standard assumption for threshold models, ensuring that values of **z** and the sign of the corresponding liabilities are in concordance.

Finally, p(**α**)  =  p(**a**|**G**)p(**β**)p(**G**)p(**R**), which follows standard assumptions, as described in the model descriptions; p(**a**|**G**) being multivariate normal, and p(**β**), p(**G**) and p(**R**) were all assigned bounded uniform priors. Additionally, **R** was restricted to be diagonal with unity diagonal elements for all liability traits, as described above. Conditional on **z**, and the restrictions mentioned above, all parameters were sampled from their full conditional densities, as in a standard multivariate linear-threshold model. The elements of **z** were sampled from the full conditional Bernoulli distributions, as described in the model section.

All models were run for 20 000 rounds of burn-in, and thereafter for 2000 000 rounds; results were stored after every 100 rounds. Convergence of the Markov chains was confirmed by visual inspection of trace plots and by Raftery and Lewis convergence diagnostics [16]. All models converged in less than 2000 000 rounds, according to the diagnostics, but, for convenience, the same length was used for all models, and long chains were used to avoid the need to extend the chain for any of the models later.

### Model comparisons

The correlation between the estimated breeding values of the survivors for growth rate and health status, respectively, obtained from the different models was used as a measure of the similarities or differences of the models for predicting the genetic merit of these traits.

The ability of predicting growth was compared for the different models through predictive cross-validation using predictive log-scores of the models [17], approximated by evaluating the models based on posterior mean parameter estimates. Specifically, growth data were separated into

$$y_{1}={\left[y_{\mathrm{Build}}{}^{\prime }\;y_{\mathrm{Pred}}{}^{\prime }\right]}^{\prime },$$

where **y**_**Build**_ is the data used to estimate model parameters (posterior means), and **y**_**Pred**_ includes the data points (growth phenotypes) to be predicted. Posterior mean predictive log-score (PMPLS) for Model *K* was then calculated as:

$$\mathrm{PMPL}S_{K}=-\sum_{y_{i}\in y_{\mathrm{Pred}}}\log\text{p}\left(\left(y_{i}\right|M_{k},{\tilde{\theta }}_{k}\right),$$

where ${\tilde{\theta }}_{k}$ is a vector of posterior means for all model parameters for model *K*. For Model 1 (*M*_*1*_), $\text{p}\left(\left(y_{i}\right|M_{1},{\tilde{\theta }}_{1}\right)$ is the probability density function of observation *i* in **y**_**Pred**_, assuming a standard Gaussian model and the parameters estimated with this model. For Model 2 (*M*_*2*_):

$$\begin{array}{ll} \;p\left(\left(y_{i}\right|M_{2},{\tilde{\theta }}_{2}\right) & =\pi_{i}\text{p}\left(\left(y_{i}\right|M_{2},{\tilde{\theta }}_{2},Z_{i}=1\right)\\ & \;+\left(1-\pi_{i}\right)\text{p}\left(\left(y_{i}\right|M_{2},{\tilde{\theta }}_{2},Z_{i}=0\right), \end{array}$$

where $\pi_{i}=\text{Pr}\left(\left(Z_{i}=1\right|{\tilde{\theta }}_{2}\right)=\Phi \left({\tilde{\lambda }}_{2i}\right)$, and ${\tilde{\lambda }}_{2i}$ is the expected liability of being healthy for observation *i*, given the associated parameters in ${\tilde{\theta }}_{2}$.

A low PMPLS indicates a better predictive ability, and models with the lowest possible PMPLS are therefore favored. A randomly chosen subset consisting of 10% of the observations was included in **y**_**Pred**_, and parameters were estimated based on the remaining 90% of the observations in **y**_**Build**_. Five different sets of **y**_**Pred**_ and **y**_**Build**_ were extracted by removing a random set of 10% of the growth records and used to estimate the predictive ability of the models.

## Results

### Phenotypic growth-distributions

**Figure 1:**
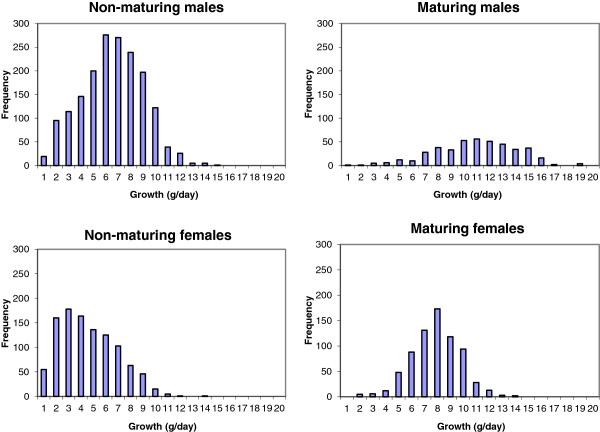
Phenotypic growth distributions for the four different sex-maturation-categories.

Figure 1 shows the frequency distribution of growth rates for animals in each of the four categories of sex and sexual maturation. Generally, the growth rate of the fish was lower than usually recorded during the periods without IPN-infections (results not shown). At the time of recording, 2.5 to 3 years after tagging, only 20% of the males and 40% of the females were maturing. The categories differed in average growth as well as in the distributions of growth. Both maturing and non-maturing males had skewed distributions resembling mixtures. The same applied to non-maturing females, while the growth distribution of maturing females was close to a normal distribution. The Jarque-Bera test [18] indicated that all four distributions in Figure 1 deviated from the normal distribution (p < 0.05), with p-values ranging from 0.031 (maturing males) to less than 0.001 (non-maturing females), i.e., the maturing males and non-maturing females were less and most deviant from a normal distribution, respectively. This may indicate a low incidence of runts among maturing females and a high incidence of runts in non-maturing females.

### Estimated proportions of healthy animals

The estimated proportions of healthy fish within each systematic effect category (Table 2) confirmed several of the differences that were observed from visual inspection of the growth distributions shown in Figure 1. Maturing females, which seemed to have a growth distribution most closely resembling a normal distribution, led to very high proportions of healthy fish in all models (ranging from 0.99-1.00). Non-maturing females showed the lowest proportion of healthy fish, which could explain their skewed phenotypic growth distribution. Also in males, the non-maturing group had the lowest proportion of healthy fish, but with a smaller difference between maturing and non-maturing groups than in females. Model 2, in which health status and sexual maturation were assumed to be independent effects, estimated a much lower infection-rate (6%) than the models that assumed that infection also affected liability for sexual maturation (15% in Models 3 and 4). Finally, the model that assumed the existence of a genetic component for growth in runts (Model 5) gave an even higher estimated occurrence of runts (19%).

**Table 2:** **Proportions of fish ± standard error^2 ^classified as healthy**

		**Males**		**Females**	
**Model^1^**	**All data**	**Non-maturing**	**Maturing**	**Non-maturing**	**Maturing**
2	0.94 ± 0.01	0.93	0.95	0.91	0.99
3	0.85 ± 0.02	0.85	0.92	0.71	1.00
4	0.85 ± 0.01	0.85	0.92	0.70	1.00
5	0.81 ± 0.02	0.81	0.90	0.65	1.00

Proportions of healthy fish were given for the whole data set and for each sex-maturation class.

^1^ Models: (2) Bivariate model including growth and liability of being healthy; (3) Extension of (2) where sexual maturation is included as a third trait; (4) An extension of (3) to also include a survival-model with genetic effects; (5) An extension of (4) to include a genetic effect of growth in runts as well as in healthy animals.

^2^ Standard errors of proportions of healthy animals were only available for the whole dataset, due to software limitations.

### Estimated systematic effects

**Figure 2:**
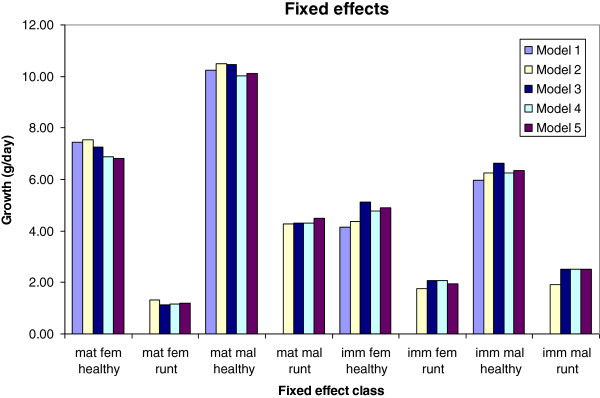
**Estimated systematic effects from five models.** (1) Classic animal model for growth; (2) Bivariate model including growth and liability of being healthy; (3) Extension of (2) where sexual maturation is included as a third trait; (4) An extension of (3) to also include a survival-model with genetic effects; (5) An extension of (4) to include a genetic effect of growth in runts as well as in healthy animals. Model (1) did not distinguish between healthy animals and runts, but its fixed effects were compared to the fixed effects obtained for healthy animals with the other models, as most animals belonged to that category, and it is common, when health is not recorded, to assume that recorded animals are healthy.

The expected growth rates within each fixed effect category, estimated by the different models, are presented in Figure 2. For runts, expected growth was estimated as the effect of sex-maturation class. For healthy fish, expected growth was estimated as the sum of the estimated effect of sex-maturation class and the effect of being healthy, nested within that sex-maturation class. It should be noted that the effect of being healthy was estimated jointly for maturing males and females. This was due to the near absence of runts within maturing females, causing the expected growth of maturing female runts to be interpreted from the corresponding difference in growth between runt and healthy maturing males. The figure shows that growth rates differ substantially between maturing and non-maturing animals, and also that the health status has a strong impact on growth rate, especially in maturing animals. There were no significant differences between the models with respect to estimates of the systematic effects.

### Heritabilities

The estimated heritabilities for the different models are reported in Table 3. The estimated heritability of growth, both in healthy fish and (when included) in runts, was consistently of medium magnitude and on average equal to 0.29 for all models. The estimated heritability for liability for health status varied between models, but was always significantly positive (0.36-0.68). Heritability of observed survival was close to 0.5, with a rather low standard error (Models 4 and 5). Finally, the estimated heritability for liability for sexual maturation was around 0.3 for all models that included this trait in the analysis.

**Table 3:** Posterior means heritabilities of the four traits, estimated with five models

**Model^1^**	**Healthy growth**	**Liability of healthy**	**Sexual maturation**	**Survival**	**Runt growth**
1	0.24 (0.03)	-	-	-	-
2	0.25 (0.03)	0.68 (0.11)	-	-	-
3	0.27 (0.04)	0.44 (0.08)	0.32 (0.04)	-	-
4	0.32 (0.04)	0.39 (0.07)	0.33 (0.05)	0.47 (0.03)	-
5	0.35 (0.05)	0.36 (0.08)	0.33 (0.05)	0.48 (0.03)	0.28 (0.11)

Posterior standard errors, estimated as standard deviations between the stored rounds of Gibbs sampler, are given in brackets.

^1^ Models: (1) Normal animal model for growth; (2) Bivariate model including growth and liability of being healthy; (3) Extension of (2) where sexual maturation is included as a third trait; (4) An extension of (3) to also include a survival-model with genetic effects; (5) An extension of (4) to include a genetic effect of growth in runts as well as in healthy animals.

### Genetic correlations

Estimated genetic correlations between the traits are reported in Table 4. The different models generally agreed on the estimated genetic correlations, but large posterior standard deviations of the sampled correlations makes it difficult to compare them across models. The genetic correlation between growth of healthy fish and liability for health status tended to be positive. However, the estimated genetic correlation between liability to be healthy and growth of runts (when included) was close to zero. Survival tended to have low to moderate favorable genetic correlations with the other traits, while there was a tendency towards weak to moderately unfavorable genetic correlations between sexual maturation and health.

**Table 4:** Posterior means for genetic correlations ± posterior standard deviations estimated with four models

	**Healthy growth**	**Health**	**Maturation**	**Survival**
Health	0.18 ± 0.18_(2)_			
	0.29 ± 0.14_(3)_			
	0.36 ± 0.13_(4)_			
	0.30 ± 0.19_(5)_			
Maturation	0.34 ± 0.11_(3)_	-0.30 ± 0.15_(3)_		
	0.37 ± 0.11_(4)_	-0.19 ± 0.12_(4)_		
	0.39 ± 0.12_(5)_	-0.16 ± 0.17_(5)_		
Survival	0.47 ± 0.08_(4)_	0.29 ± 0.13_(4)_	0.19 ± 0.12_(4)_	
	0.50 ± 0.09_(5)_	0.18 ± 0.16_(5)_	0.22 ± 0.12_(5)_	
Runt growth	0.55 ± 0.17_(5)_	0.06 ± 0.31_(5)_	0.08 ± 0.25_(5)_	0.58 ± 0.21_(5)_

Posterior standard deviations are the standard deviations between the stored rounds of the Gibbs sampler. The model^1^ is included as a subscript.

^1^ Models: (2) Bivariate model including growth and liability of being healthy; (3) Extension of (2) where sexual maturation is included as a third trait; (4) An extension of (3) to also include a survival-model with genetic effects; (5) An extension of (4) to include a genetic effect of growth in runts as well as in healthy animals.

### Correlations between breeding values obtained by different models

Table 5 shows that high correlations were observed between breeding values obtained with models that included the same traits (i.e. between model 1 and model 2 and between model 4 and model 5). Correlations between models that included different traits were somewhat lower, ranging from 0.82 to 0.94, which was expected since the traits included in the study were correlated.

**Table 5:** Correlations between estimated breeding values ± their standard errors for growth, obtained with four models

**Model^1^**	**1**	**2**	**3**	**4**
2	0.97 ± 0.002			
3	0.89 ± 0.004	0.94 ± 0.003		
4	0.83 ± 0.005	0.86 ± 0.004	0.91 ± 0.003	
5	0.82 ± 0.005	0.84 ± 0.004	0.88 ± 0.004	0.99 ± 0.001

^1^ Models: (2) Bivariate model including growth and liability of being healthy; (3) Extension of (2) where sexual maturation is included as a third trait; (4) An extension of (3) to also include a survival-model with genetic effects; (5) An extension of (4) to include a genetic effect of growth in runts as well as in healthy animals.

The correlations between the estimated breeding values for health status, obtained by the different models, were also generally high (Table 6). The lowest correlation and thus the highest re-ranking was observed between Model 2 and the other mixture models, indicating that the health status was judged differently when assumed to affect both growth and sexual maturation.

**Table 6:** Correlations ± standard errors between estimated breeding values from three models for liability of being healthy

**Model^1^**	**2**	**3**	**4**
3	0.85 ± 0.004		
4	0.79 ± 0.005	0.88 ± 0.004	
5	0.79 ± 0.005	0.93 ± 0.003	0.93 ± 0.003

^1^ Models: (3) Multivariate mixture model including the traits growth, liability of being healthy and sexual maturation; (4) An extension of (3) to also include a survival-model with genetic effects; (5) An extension of (4) to include a genetic effect of growth in runts as well as in healthy animals.

### Model comparison

Posterior mean predictive log-scores of Models 1 and 2 differed by 88 to 142 for the five replicates (Table 7), in favor of Model 2 for all sub-sets. Hence, the results strongly indicate that a mixture model predicts novel observations systematically more accurately than a classical linear model. All mixture models consistently outperformed the classical model, but the comparisons between mixture models were less conclusive, giving small differences between the models and different ranking of the models between the different subsets (Table 7).

**Table 7:** Posterior mean predictive log-scores for growth from five models for five subsets of data

**Subset**	**Model 1^1^**	**Model 2^1^**	**Model 3^1^**	**Model 4^1^**	**Model 5^1^**
1	907.3	787.0	785.1	784.2	787.9
2	950.8	843.0	852.7	841.4	849.8
3	893.6	803.9	794.5	798.2	796.8
4	932.5	790.6	802.5	803.4	806.4
5	932.7	844.9	835.7	833.4	845.2

^1^ Models: (1) Normal animal model for growth; (2) Bivariate model including growth and liability of being healthy; (3) Extension of (2) where sexual maturation is included as a third trait; (4) An extension of (3) to also include a survival-model with genetic effects; (5) An extension of (4) to include a genetic effect of growth in runts as well as in healthy animals.

## Discussion

In this data, fish surviving the IPN outbreak had low and skewed growth distributions. Furthermore, an unusually large fraction of non-maturing fish, especially among males, was observed. A possible explanation for these results may be that the disease not only affected survival, but that health status differed among survivors and potentially had an impact on the growth rate and the reproductive development of the fish. Mixture models were used to identify such animals (runts) based on their observed growth rates and sexual maturation.

This study used the liability-normal mixture model developed by Ødegård et al. [10]. Within the field of animal breeding, this methodology has been applied to somatic cell score data of dairy cows, with the aim to identify unobserved cases of subclinical mastitis [19], and also to estimate breeding values for resistance to unobserved subclinical mastitis [11]. In this study, we used this methodology to infer health status based on observed growth rates in salmon in a multiple-trait analysis. Furthermore, some of the models were extended to involve a mixture of probits [13] with respect to sexual maturity, i.e., assuming that the unobserved health status affected both the observed growth and the liability for sexual maturation.

The mixture models applied in this study seemed to fit the data well, as indicated by the improved predictive ability of the mixture models compared to a standard classical model (Table 7). The estimated heritabilities for liability for survival in the field (IPN) ranged from 0.47 to 0.48, similar to earlier reported estimates for IPN survival (in both challenge and field tests) [1-3]. Heritabilities for (healthy) growth rate, ranging from 0.25 to 0.35, and sexual maturation (at normal age), ranging from 0.32 to 0.33, were also similar to other reported estimates [20,21]. Wild et al. [22] reported lower heritability estimates (on the liability scale) for early sexual maturity across several test farms (0.10 and 0.17 for the two studied year-classes and derived from the sire component of variance), which may be an effect of the significant genotype by test farm interaction that they identified. Our positive genetic correlations between growth rate and normal sexual maturation (0.34-0.39) are higher than the estimates reported by Gjerde et al. [18] but of the same magnitude as the estimates between growth and early sexual maturity reported by Gjerde and Gjedrem [23]. Comparisons of the different mixture models, including different traits, through their ability to predict growth, showed small and inconsistent differences between the models. The small differences between Models 2, 3 and 4 indicate that inclusion of other traits had only minor effects on the ability to predict growth. However, since predictive abilities with respect to the other traits (survival and sexual maturation) were not included, this only shows a part of the full picture. The comparison of Models 4 and 5, which included the same traits, was slightly in favor of Model 4 in four out of the five subsets of data, suggesting that assuming no genetic variance in runt growth might be desirable, but this did not have large effects on the predictive ability of the model.

The current models did not include any effect common to full-sibs. This type of effect may arise from the separate rearing of the full-sib families until tagging (as well as potential non-additive genetic effects), and, if present, may bias the estimated heritability upwards. However, a preliminary analysis using standard (non-mixture) linear models for growth with ASREML [24], estimated a common environmental variance of only 2% of the genetic variance, and a likelihood ratio test of a growth model with and without a common environmental effect for full-sibs in the model gave a test statistic of 0. The common environmental effect was thus removed from the final models to prevent over-parameterization of the models. If common environmental effects existed, although not detected in the preliminary analyses, genetic variances may be over-estimated, but this bias seems small, since parameter estimates fit well with those found in the literature.

The different models were inconsistent in the estimated proportions of runts. The simplest mixture model, in which health status was assumed to affect growth only (Model 2), indicated that 6% of the surviving fish were runts (Table 2). This was an unexpectedly low proportion, judged by the skewed distributions of growth rate (Figure 1) and the low levels of growth and sexual maturation, as well as the knowledge that IPN could affect growth [7] and that this disease was probably the main cause of the high mortality (72%) in this population. Preliminary analyses applying mixture models without including sexual maturation as a fixed effect (results not shown), gave about 13% runts. Hence, sexual maturation and health status appeared to be confounded and may thus pick up some of the same effects. If sexual maturation was affected by health status, it may explain both the confounding of maturation and health effects and the different shapes of the growth distributions for maturing and non-maturing fish. Model 3 and extensions thereof (mixtures of probits) appeared to be able to handle a situation where the disease affected both sexual maturation and growth rate. With these models, a much higher proportion of the fish was classified as runts (Table 2), especially among the non-maturing animals. A single-trait mixture model for maturation would not be able to distinguish between the effects of health and sexual maturation (i.e., the binary sexual maturation status being affected by the binary and unobserved health status). Identification of runts in the proposed models is facilitated by the assumption that occurrence of infection gives a simultaneous unfavorable effect on both observed growth and liability for sexual maturation. However, the models did not have much power to distinguish between the effects of health and sexual maturation, and the apparent negative genetic correlation between these traits may be a result of a partial confounding of these effects, even when including all the relevant traits in the analysis.

The other factor that affected the estimated proportions of healthy animals was whether a genetic effect of growth in runts was included or not. This implies that being infected does not necessarily result in the same reduction in growth for all animals, i.e. such models may capture genetic variation in disease tolerance [25]. Theoretically, it seems restrictive to assume that only growth in healthy animals is affected by genetics, and our results appeared valid, even when an additive genetic effect specific to growth of runts was included. However, there are some interpretation issues with respect to the genetics of runt growth, because it changes the definition of runt from a clearly defined situation where putative runts show poor growth irrespective of their genetic growth potential, to a less clear defined situation where growth is more or less reduced (depending on the breeding value for growth in the runt vs. breeding value of the same animal when considered healthy). This change of definition led to more fish defined as runts. The weak estimated genetic correlation between growth in runts and growth in healthy fish indicates that growth in runts is a complex trait that may be affected by other factors besides genetic growth potential, possibly related to disease tolerance. In this context, tolerance should be interpreted as the severity of the symptoms after infection, while resistance describes the risk of getting infected. The genetic correlation between health, which can thus be interpreted as a measurement of resistance, and runt growth, which can be seen as a measurement of tolerance, was low, indicating that resistance and tolerance are different traits. Both these traits were however positively correlated to survival, indicating that resistance against a fatal infection has an influence on both the risk of being affected if one has survived and the severity of the symptoms if affected. Hence, breeding for survival will probably alter both resistance and tolerance to the disease. Still, extending the model to also include a runt-specific genetic effect on growth appeared to reduce the predictive ability of the model (4 vs. 5) with respect to growth. Hence, these results should be interpreted with care.

The proposed mixture models could be useful to model growth in disease-infested environments. The method gives consistent estimates of heritabilities and genetic correlations between traits, and makes it possible to estimate breeding values for health, or environmental robustness, that might be more informative than a standard survival analysis based on challenge tests only. Furthermore, the mixture model (Model 2) had a consistently higher predictive ability than the conventional non-mixture model (Model 1), indicating that mixture models are more appropriate to analyse this type of data. However, the low proportion of maturing fish, the low average growth and the large deviations of phenotypic growth distributions from the normal distribution cannot be explained by the occurrence of only 5% runts, as indicated by Model 2. It is therefore reasonable to believe that the more complex models that yield higher proportions of runts also have fewer misclassifications, and probably have greater predictive ability than the simpler Model 2.

In the case of IPN in Atlantic salmon, runts may not only occur as a direct effect of IPN, but may also occur as an indirect effect of secondary infections. Thus, health status (runt/healthy) and IPN resistance may be partially different traits. This may explain the rather low genetic correlation between liability to being healthy and liability for survival. In a scenario like this, additional information obtained by the mixture models can be of particular importance as a supplement to challenge test data, e.g., if the aim is to model not only the risk of dying from the infection, but also the risk of secondary problems among the survivors. Furthermore, the method may be especially relevant for non-lethal diseases/conditions that cause losses in terms of growth (or other indicator traits), or if the pathogenic load has to be increased to unrealistic levels to be able to observe mortality (e.g., pancreas disease in salmon). It may also be possible to use data from natural disease outbreaks more efficiently.

## Competing interests

The authors declare that they have no competing interests.

## Authors’ contributions

ML carried out the statistical analyses, participated in developing the statistical models and drafted parts of the manuscript. JØ planned the study, edited the data, participated in developing the statistical models and modifications of the statistical software and drafted parts of the manuscript. PM modified the statistical software to handle complex mixture models. BG contributed with knowledge about salmon and IPN. TR participated in describing the data and with knowledge about salmon and IPN in general. MR participated in planning the study, coordinated the project and contributed with knowledge about salmon and IPN. All authors participated in the discussion and revisions of the manuscript and read and approved the final manuscript.

## References

[B1] Drangsholt TM Gjerde B Ødegård J Finne-Fridell F Evensen O Bentsen HB Quantitative genetics of disease resistance in vaccinated and unvaccinated Atlantic salmon (Salmo salar L.) Heredity 2011 107 471 477 10.1038/hdy.2011.34 21559049 PMC3199929

[B2] Guy DR Bishop SC Brotherstone S Hamilton A Roberts RJ McAndrew BJ Woolliams JA Analysis of the incidence of infectious pancreatic necrosis mortality in pedigreed Atlantic salmon, Salmo salar L., populations J Fish Dis 2006 29 637 647 10.1111/j.1365-2761.2006.00758.x 17169110

[B3] Wetten M Aasmundstad T Kjøglum S Storset A Genetic analysis of resistance to infectious pancreatic necrosis in Atlantic salmon (Salmo salar L.) Aquaculture 2007 272 111 117 10.1016/j.aquaculture.2007.08.046

[B4] Moen T Baranski M Sonesson AK Kjøglum S Confirmation and fine-mapping of a major QTL for resistance to infectious pancreatic necrosis in Atlantic salmon (*Salmo salar*): population-level associations between markers and trait BMC Genomics 2009 10 368 10.1186/1471-2164-10-368 19664221 PMC2728743

[B5] Houston RD Haley CS Hamilton A Guy DR Mota-Velasco JC Gheyas AA Tinch AE Taggart JB Bron JE Starkey WG McAndrew BJ Verner-Jeffreys DW Paley RK Rimmer GSE Tew IJ Bishop SC The susceptibility of Atlantic salmon fry to freshwater infectious pancreatic necrosis is largely explained by a major QTL Heredity 2010 105 318 327 10.1038/hdy.2009.171 19935825

[B6] Bornø G Sviland C Farmed fish health report 2010 2010 http://www.vetinst.no/eng/Research/Publications/Fish-Health-Report/Farmed-Fish-Healt-Report-2010

[B7] Damsgård B Mortensen A Sommer AI Effects of infectious pancreatic necrosis virus (IPNV) on appetite and growth in Atlantic salmon, Salmo salar L Aquaculture 1998 163 185 193 10.1016/S0044-8486(98)00242-7

[B8] McLachlan G Peel D Finite mixture models 2000 New York: Wiley

[B9] Ødegård J Jensen J Madsen P Gianola D Klemetsdal G Heringstad B Detection of mastitis in dairy cattle by use of mixture models for repeated somatic cell scores: a Bayesian approach via Gibbs sampling J Dairy Sci 2003 86 3694 3703 10.3168/jds.S0022-0302(03)73975-7 14672200

[B10] Ødegård J Madsen P Gianola D Klemetsdal G Jensen J Heringstad B Korsgaard IR A Bayesian threshold-normal mixture model for analysis of a continuous mastitis-related trait J Dairy Sci 2005 88 2652 2659 10.3168/jds.S0022-0302(05)72942-8 15956327

[B11] Madsen P Shariati MM Ødegård J Genetic analysis of somatic cell score in Danish Holsteins using a liability-normal mixture model J Dairy Sci 2008 91 4355 4364 10.3168/jds.2008-1128 18946141

[B12] Gianola D Ødegård J Heringstad B Klemetsdal G Sorensen D Madsen P Jensen J Detilleux J Mixture model for inferring susceptibility to mastitis in dairy cattle: a procedure for likelihood-based inference Genet Sel Evol 2004 36 3 27 10.1186/1297-9686-36-1-3 14713407 PMC2697178

[B13] Lwin T Martin PJ Probits of mixtures Biometrics 1989 45 721 732 10.2307/2531679 2790119

[B14] Madsen P Jensen J DMU: a user's guide. A package for analysing multivariate mixed models. Version 6, release 5.0, University of Aarhus, Faculty of Agricultural Sciences (DJF) 2010 Tjele, Denmark: Department of Genetics and Biotechnology

[B15] Ødegård J Meuwissen THE Heringstad B Madsen P A simple algorithm to estimate genetic variance in an animal threshold model using Bayesian inference Genet Sel Evol 2010 42 29 10.1186/1297-9686-42-29 20649962 PMC2918534

[B16] Raftery AE Lewis SM Comment: one long run with diagnostics: implementation strategies for Markov chain Monte Carlo Stat Sci 1992 7 493 497 10.1214/ss/1177011143

[B17] Sorensen D Gianola D Likelihood, Bayesian and MCMC methods in quantitative genetics 2002 New York: Springer

[B18] Jarque CM Bera AK A test for normality of observations and regression residuals Int Stat Rev 1987 55 163 172 10.2307/1403192

[B19] Boettcher PJ Caraviello D Gianola D Genetic analysis of somatic cell scores in US Holsteins with a Bayesian mixture model J Dairy Sci 2007 90 435 443 10.3168/jds.S0022-0302(07)72645-0 17183112

[B20] Gjedrem T Genetic improvement of cold-water fish species Aquac Res 2000 31 25 33 10.1046/j.1365-2109.2000.00389.x

[B21] Gjerde B Simianer H Refstie T Estimates of genetic and phenotypic parameters for body weight, growth rate and sexual maturity in Atlantic salmon Livest Prod Sci 1994 38 133 143 10.1016/0301-6226(94)90057-4

[B22] Wild V Simianer H Gjoen HM Gjerde B Genetic parameters and genotype X environment interaction for early sexual maturity in Atlantic salmon (*Salmo salar*) Aquaculture 1994 128 51 65 10.1016/0044-8486(94)90101-5

[B23] Gjerde B Gjedrem T Estimates of phenotypic and genetic parameters for carcass traits in Atlantic salmon and rainbow trout Aquaculture 1984 36 97 110 10.1016/0044-8486(84)90057-7

[B24] Gilmour AR Gogel BJ Cullis BR Thompson R ASReml user guide release 3.0 2009 Hemel Hempstead: VSN International Ltd

[B25] Kause A Genetic analysis of tolerance to infections using random regressions: a simulation study Genet Res 2011 93 291 302 10.1017/S0016672311000176 21767462

